# YL064 directly inhibits STAT3 activity to induce apoptosis of multiple myeloma cells

**DOI:** 10.1038/s41420-018-0108-8

**Published:** 2018-10-03

**Authors:** Yingying Wang, Linlin Wu, Haiyan Cai, Hu Lei, Chun-Min Ma, Li Yang, Hanzhang Xu, Qi Zhu, Zhujun Yao, Yingli Wu

**Affiliations:** 1 Hongqiao International Institute of Medicine, Shanghai Tongren Hospital/Faculty of Basic Medicine, Chemical Biology Division of Shanghai Universities E-Institutes, Key Laboratory of Cell Differentiation and Apoptosis of the Chinese Ministry of Education, Shanghai Jiao Tong University School of Medicine, 200025 Shanghai, China; 2 Department of Hematology, Shanghai 9th People’s Hospital, Shanghai Jiao Tong University School of Medicine, 639 Zhizaoju Road, 200011 Shanghai, China; 3 State Key Laboratory of Coordination Chemistry, Jiangsu Key Laboratory of Advanced Organic Materials, School of Chemistry and Chemical Engineering, Nanjing University, 163 Xianlin Avenue, 210023 Nanjing, Jiangsu China

**Keywords:** Apoptosis, Myeloma

## Abstract

Aberrant activation of signal transducer and activator of transcription 3 (STAT3) plays a critical role in the proliferation and survival of multiple myeloma. And inactivation of STAT3 is considered a promising strategy for the treatment of multiple myeloma. Here we show that the sinomenine derivative YL064 could selectively reduce the cell viability of multiple myeloma cell lines and primary multiple myeloma cells. Moreover, YL064 also induces cell death of myeloma cells in the presence of stromal cells. Western blot analysis showed that YL064 inhibited the constitutive activation and IL-6-induced activation of STAT3, reflected by the decreased phosphorylation of STAT3 on Tyr705. Consistent with this, YL064 inhibited the nuclear translocation of STAT3 and the expression of STAT3 target genes, such as cyclin D1 and Mcl-1. Using biotin- and FITC-labeled YL064, we found that YL064 could pull-down STAT3 from myeloma cells and colocalized with STAT3, suggesting that YL064 directly targets STAT3. Cellular thermal shift assay further demonstrated the engagement of YL064 to STAT3 in cells. Molecular docking studies indicated that YL064 may interact with STAT3 in its SH2 domain, thereby inhibiting the dimerization of STAT3. Finally, YL064 inhibited the growth of human myeloma xenograft in vivo. Taken together, this study demonstrated that YL064 may be a promising candidate compound for the treatment of multiple myeloma by directly targeting STAT3.

## Introduction

Multiple myeloma (MM) accounts for approximately 1% of all malignancies^1^. Though the advent of bortezomib represents a promising strategy for MM treatment^2^, the development of off-target toxicities and drug resistance limit its effectiveness^3^. Therefore, identifying novel therapeutic approaches are critical medical needs.

Signal transducer and activator of transcription 3 (STAT3) is a multi-functional factor and is important in regulating cell proliferation, differentiation, survival, and inflammatory response^4^. The activation of STAT3 augments the expression of numerous genes, such as Bcl-xL, Mcl-1, cyclin D1, and vascular endothelial growth factor, which could enhance cancer cell survival or proliferation^5–7^. In various solid tumors and hematological malignancies, cytokine and growth factor receptors are constitutively secreted or expressed. In response to cytokine-receptor binding (such as interlukin-6 (IL-6)), STAT3 was activated by tyrosine phosphorylation (Tyr705) and dimerization, followed by nuclear translocation and regulating the expression of its target genes^8–11^. Of note, constitutive activation STAT3 may play a more crucial role in the pathogenesis of MM. In the bone marrow environment, cytokines such as IL-6, secreted by stromal cells or the myeloma cells, could lead to the constitutive activation of STAT3. Consequently, targeting STAT3 may be a promising strategy to combat MM^12^.

Sinomeniumacutum, a Chinese medical plant, has been used for the treatment of various rheumatic diseases in China for over 2000 years^13^. Sinomenine, a component isolated from Sinomeniumacutum, has been used to treat rheumatic diseases including rheumatoid arthritis (RA)^14^. However, the clinical use of sinomenine is limited, because it has to be used at high dose, which lead to obvious side effects. Therefore, a number of sinomenine derivatives were synthesized to minimize side effects and improve its efficacy^15^. In the present study, we demonstrate that YL064, a novel sinomenine derivative, could directly inhibit STAT3 activation and induce cell death in myeloma cells both in vitro and in vivo.

## Results

### YL064 selectively induces apoptosis in primary and MM cell lines

**Fig. 1:**
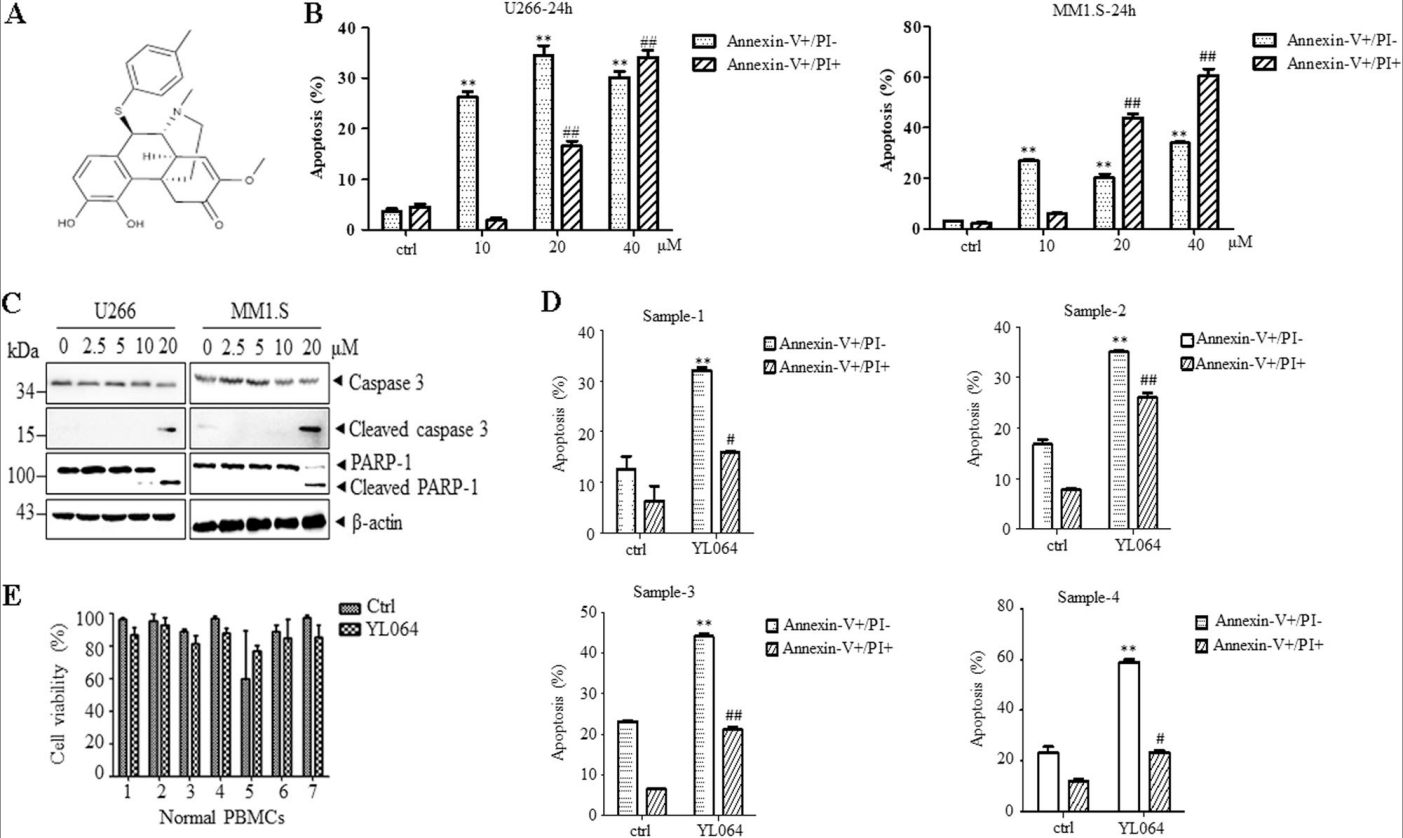
YL064 selectively induces myeloma cell death. **a** Chemical structure of YL064. **b** U266 and MM1.S cells were treated with the indicated concentrations of YL064 for 24 h and apoptotic cells were evaluated by Annexin V/PI double-staining assay. **p < 0.01, compared with control annexin V+/PI– cells, ^##^*p* < 0.01, compared with control annexin V+/PI+ cells. **c** U266 and MM1.S cells were untreated or treated with indicated concentrations of YL064 for 24 h. The indicated proteins were detected by western blot. **d** Primary CD138^+^ MM cells were treated with YL064 (20 μM) for 24 h and CD138^+^ apoptotic cells were evaluated by Annexin V/PI double-staining assay. **p < 0.01, compared with control annexin V+/PI– cells, ^#^*p* < 0.05, ^##^*p* < 0.01, compared with control annexin V+/PI+ cells. **e** Primary PBMCs cells from healthy donors were treated with the indicated concentrations of YL064 (40 μM) for 24 h and cell viability was evaluated by Trypan blue exclusion assay

The cytotoxic effects of YL064 were evaluated on MM cell lines and CD138-positive primary MM cells. YL064 (Fig. 1a) induces apoptosis of U266 and MM1.S cells in a time- and dose-dependent manner (Fig. 1b), as reflected by the cleavage of caspase-3 and Poly [ADP-ribose] polymerase 1 (Fig. 1c). Next, we examined the effect of YL064 on primary CD138-positive cells. The results showed that YL064 significantly induced cell death of them (Fig. 1d). However, even at 40 μM, YL064 did not change the cell viability of normal human peripheral blood mononuclear cells (PBMCs; Fig. 1e). These results suggest that YL064 could selectively induce MM but not PBMCs cell death.

### YL064 inhibited STAT3 phosphorylation at Tyr705 in MM cells

**Fig. 2:**
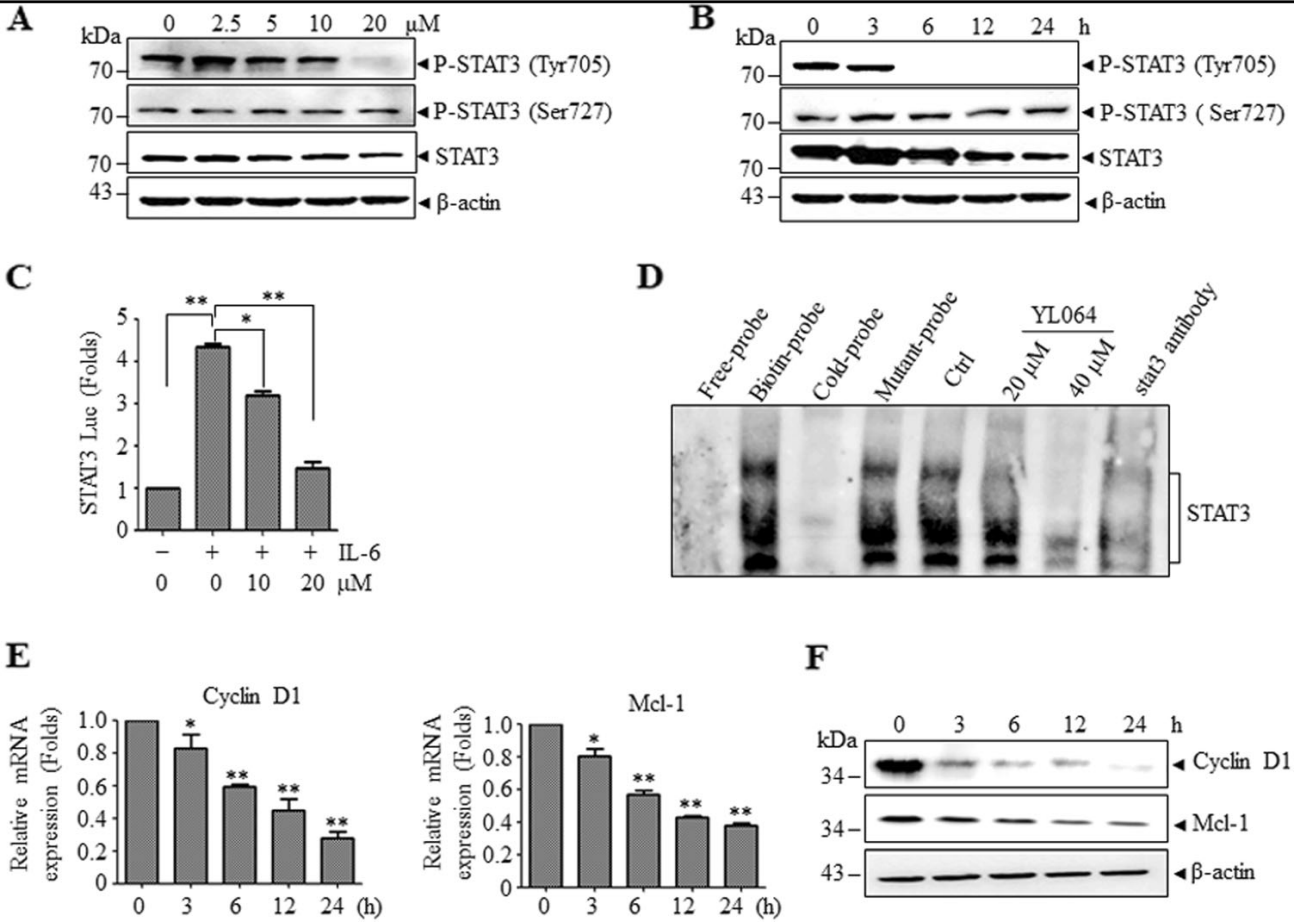
YL064 inhibits constitutively active STAT3. **a,** **b** U266 cells were treated with the indicated concentrations of YL064 for 24 h (**a**) or treated with YL064 (20 μM) for different times (**b**). Cells were collected and subjected to western blot analyses with specific antibodies against P-STAT3 (Tyr705), P-STAT3 (Ser727), and STAT3. **c** HeLa cells were transfected with p-STAT3-Luc and SV40-Renilla-Luc. Then, cells were treated with YL064 (20 μM) for 24 h, followed by stimulating with IL-6 for 20 min, and the luciferase activity were measured *p < 0.05, **p < 0.01. **d** U266 cells were treated with the indicated concentrations of YL064 for 6 h and STAT3 activity was examined by EMSA. **e,** **f** U266 cells were treated with YL064 (20 μM) for the indicated time points, and the mRNA level of cyclin D1, Mcl-1 were examined by RT-PCR and the indicated proteins were examined by western blot *p < 0.05, **p < 0.01

As STAT3 is constitutively activated and is essential for the proliferation and survival of U266 cells and YL064 selectively inhibited the proliferation of U266 cells, we speculated that the activity of STAT3 may be affected by YL064. As shown in Fig. 2a, the phosphorylation of STAT3 at Tyr705 was inhibited by YL064, whereas, the phosphorylation at Ser727 was not affected. Moreover, the phosphorylation of STAT3 at Tyr705 was completely abrogated as early as 6 h upon treatment with YL064 (Fig. 2b). In line with these findings, p-STAT3-luciferase (Luc) reporter assay revealed that IL-6-stimulated STAT3 activation was significantly diminished upon YL064 treatment (Fig. 2c). Electrophoresis mobility shift assay (EMSA) analysis revealed that YL064 decreased STAT3 DNA-binding activity (Fig. 2d), Consistent with these results, the expression of the downstream genes of STAT3, such as cyclin D1 and Mcl-1, was suppressed by YL064 both at mRNA (Fig. 2e) and protein (Fig. 2f) levels.

**Fig. 3:**
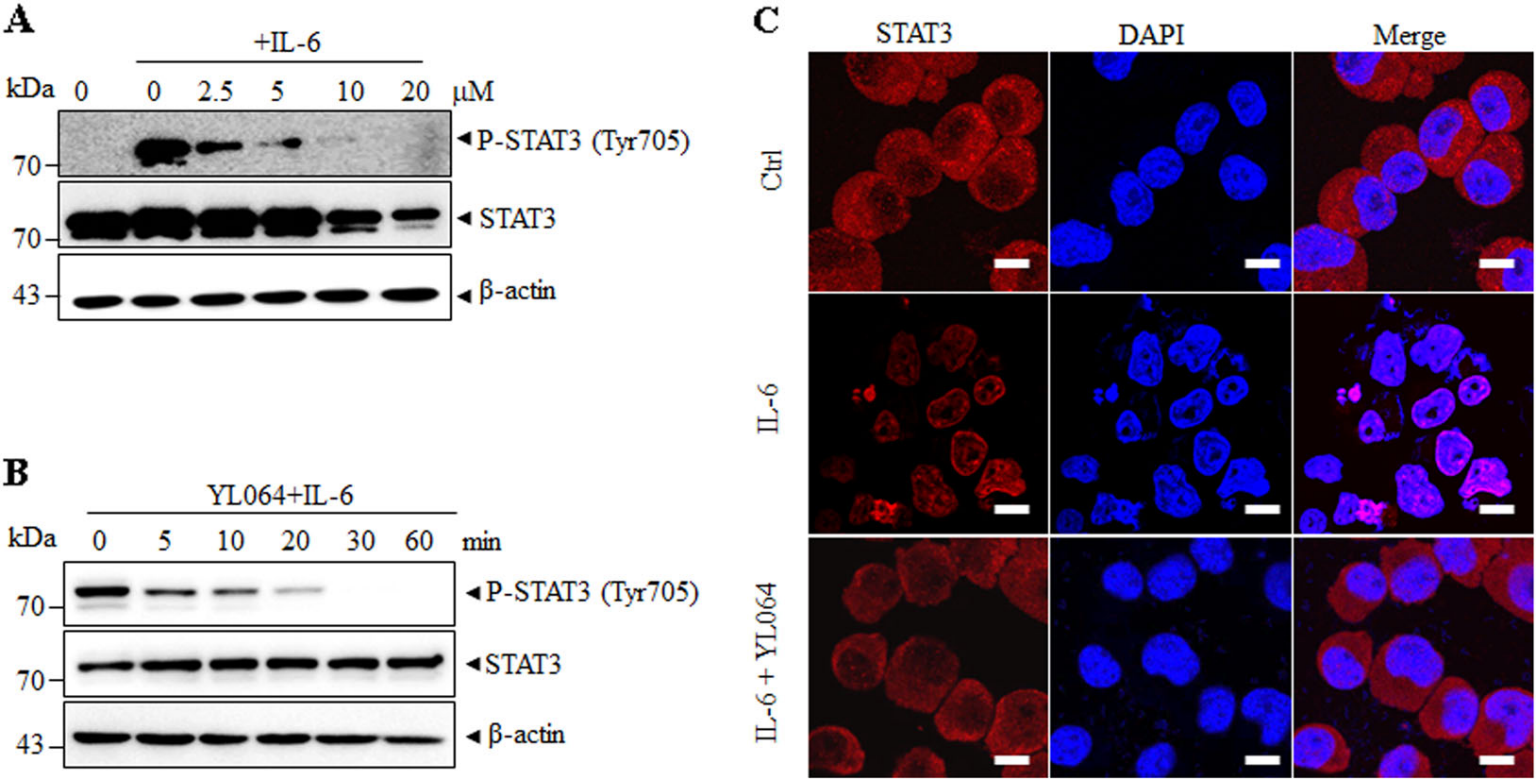
YL064 inhibits IL-6-induced phosphorylation of STAT3. **a**, **b** MM1.S cells were treated with the indicated concentrations of YL064 for 6 h (**a**) or YL064 at 20 μM for different times (**b**). The indicated proteins were detected by western blot analysis. **c** MM1.S cells were incubated with or without 20 μM YL064 for 6 h and then the intracellular distribution of STAT3 was analyzed by immunofluorescence

To examine whether the above observed phenomenon is not limited to U266 cells, we next treated IL-6-stimulated MM1.S cells with YL064. IL-6 could enhance STAT3 phosphorylation in MM1.S (Figs. 3a, b). Intriguingly, the STAT3 phosphorylation was inhibited after the exposure of YL064 for 1 h (Fig. 3b). As STAT3 phosphorylation is essential for its nuclear translocation, we then evaluated the effect of YL064 on the intracellular localization of STAT3. Immunofluorescence staining suggested that IL-6-induced nuclear translocation of STAT3 was blocked by YL064 (Fig. 3c). These data demonstrate that YL064 could abrogate STAT3 activity in MM cells.

### YL064 exhibits cytotoxic effects in bone marrow stromal cells co-cultured MM cells

**Fig. 4:**
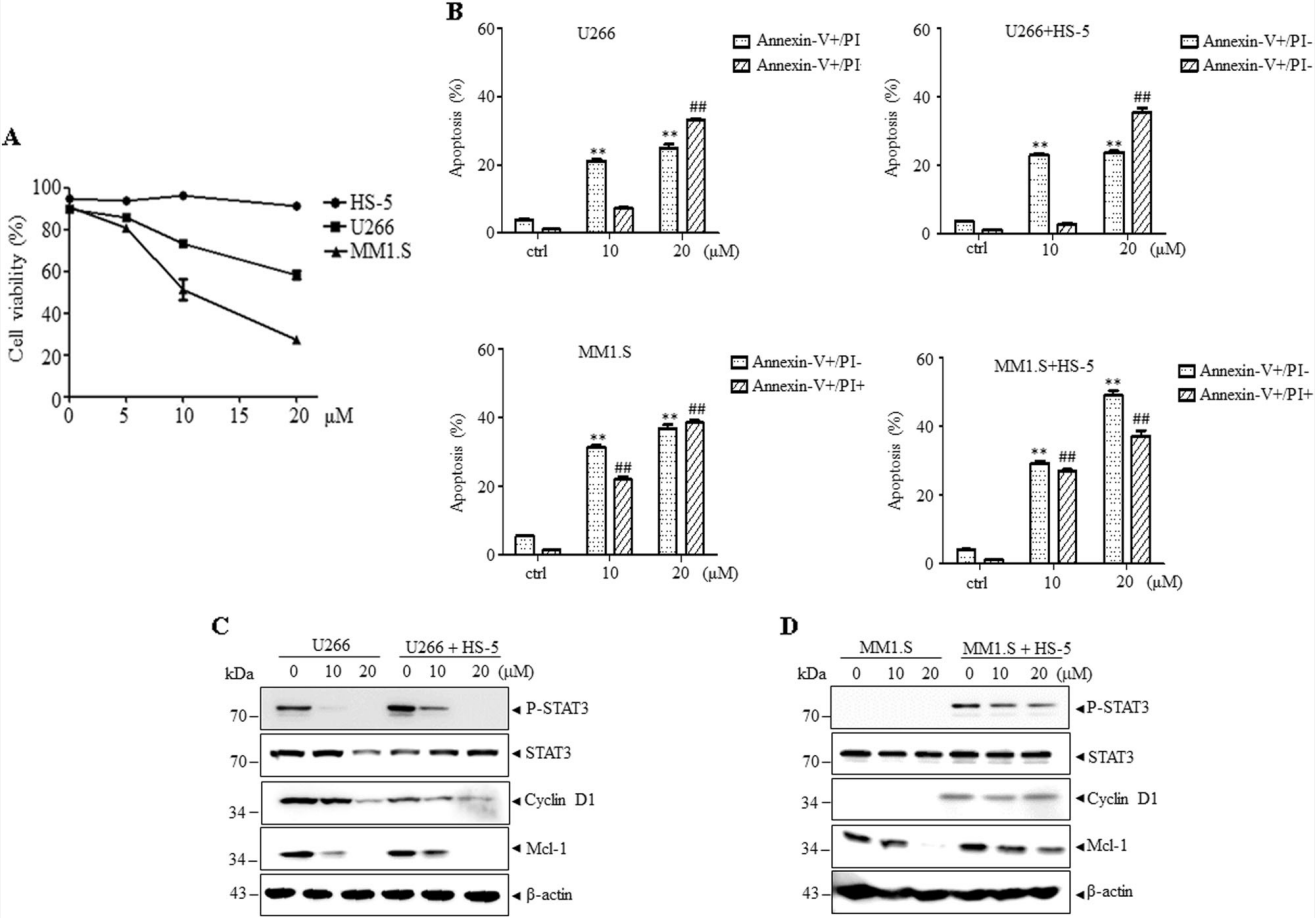
YL064 inhibits HS-5 co-culture-induced activation of STAT3 in myeloma cells. **a** HS-5, U266, and MM1.S cells were treated with the indicated concentrations of YL064 for 24 h. Cell viability was determined by Trypan blue assay. Values represented as graphs are the mean of three independent experiments with the standard deviation. **b**-**d** U266 and MM1.S cells were seeded on an established HS-5 stromal layer for 24 h, then the cells were treated with YL064 (20 μM) for additional 24 h. Subsequently, cells were separated from the HS-5 stromal layer, and apoptotic cells were examined by Annexin V/PI double-staining (**b**). **p < 0.01, compared with control annexin V+/PI– cells, ^##^*p* < 0.01, compared with control annexin V+/PI+ cells. The indicated proteins were examined by western blot (**c**, **d**)

Bone marrow stromal cells (BMSCs) were reported to protect myeloma cells from cytostatic compounds through activating STAT3 cascade^16,17^. As shown in Fig. 4a, the cell viability of U266 or MM1.S cells was decreased by YL064, whereas the HS-5 cells were not affected. When U266 or MM1.S cells were co-cultured with HS-5 cell, YL064 still exerts cytotoxic effects on these cells though the effects were diminished (Fig. 4b). This may be explained by the fact that co-culturing U266 or MM1.S cells with human bone marrow stromal-derived HS-5 cells line triggered STAT3 activation (Figs. 4c, d). However, the activation of STAT3 could also be inhibited in different degree by YL064 and thus the expression of Mcl-1 and cyclin D1 was also decreased (Figs. 4c, d). These data suggest that YL064 exhibits cytotoxic effects in BMSCs co-cultured MM cells.

### YL064 directly interacting with STAT3 in MM cells

**Fig. 5:**
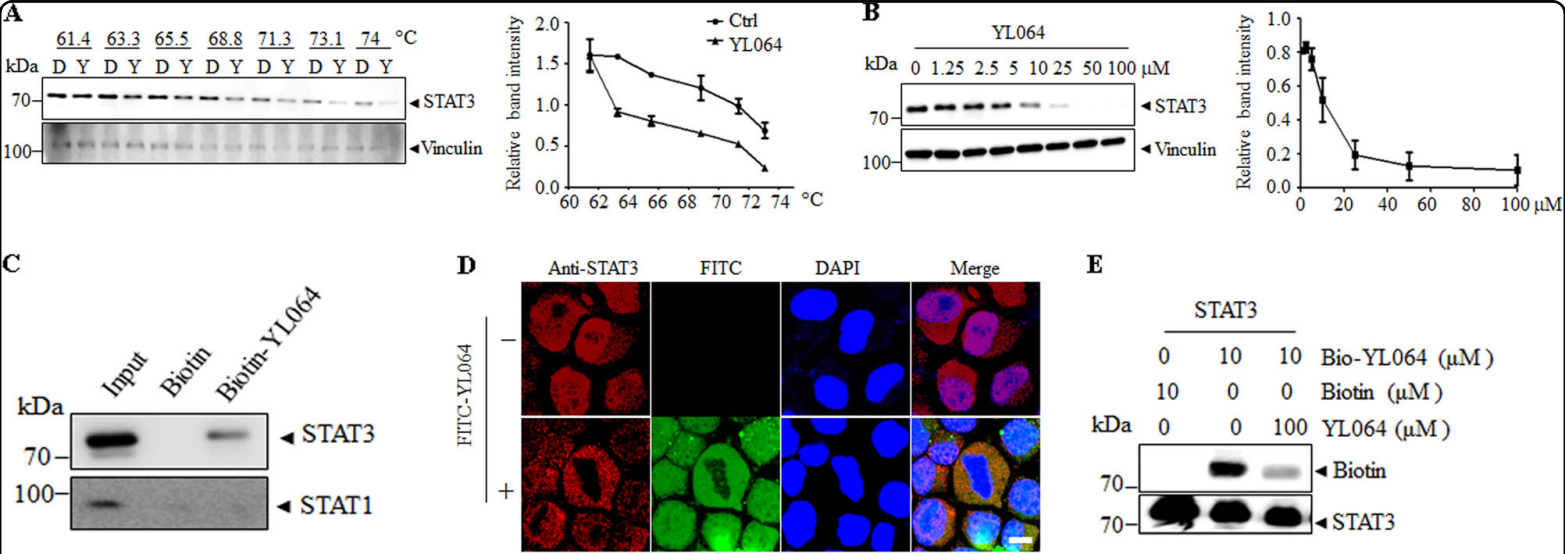
YL064 interacts with STAT3 in cells. **a** CETSA was performed on U266 cells as described in Materials and methods section. The thermal stability of STAT3 in cell lysate treated with YL064 (100 μM) at different temperatures (**a**) or different doses (**b**). The indicated proteins were evaluated by western blot. The intensity of the STAT3 bands was quantified by Quantity One software. Each experiment was repeated as least three times. **c** U266 cell lysate was incubated with biotin-YL064 (100 μM) or biotin for followed by pull-down with streptavidin-agarose beads. The precipitates were examined by western blot against STAT3 and STAT1. **d** U266 cells were treated with Fluorescein isothiocyanate (FITC) or 20 μM FITC-YL064 for 8 h, and then the colocalization of STAT3 (red) and YL064 (green) were examined by immunofluorescence staining. **e** The recombinant STAT3 protein was incubated with biotin-YL064 in the absence or presence of a ten-fold excess of unlabeled YL064 for 2 h, and the mixtures were blotted for biotin and STAT3

As YL064 could quickly inhibit the activation of STAT3 in different cells, we assumed that YL064 may directly act on STAT3. To test this possibility, we performed cellular thermal shift assay (CETSA). As shown in Figs. 5a, b, compared with dimethylsulfoxide (DMSO), YL064 markedly lowered the thermal stability of STAT3, indicating the direct engagement of YL064 with STAT3. To further validate this phenomenon, biotin- and FITC-labeled YL064 were synthesized (Supplementary Fig. S1a, 1c). The labeled YL064 also inhibits the phosphorylation of STAT3 (Supplementary Fig. S1b, 1d), indicating the modification did not change the STAT3 inhibition activity of YL064. The U266 cell lysates were incubated with biotin-YL064 or free biotin, and then streptavidin coupled beads were used to pull-down the biotins. Of note, biotin-YL064 but not biotin pulled down STAT3 (Fig. 5c) from the cell lysates. As expected, neither biotin-YL064 nor biotin could pull-down STAT1. Furthermore, FITC-YL064 was used to indicate localization of YL064 in cells. Immunofluorescence staining indicated that YL064 colocalized with STAT3 in U266 cells (Fig. 5d). In line with these observations, the interaction between recombinant STAT3 and biotin-YL064 could be competitively inhibited by unlabeled YL064 (Fig. 5e). Taken together, these data suggest that YL064 directly interacts with STAT3 in MM cells.

### SH2 domain of STAT3 may be the binding site for YL064

**Fig. 6:**
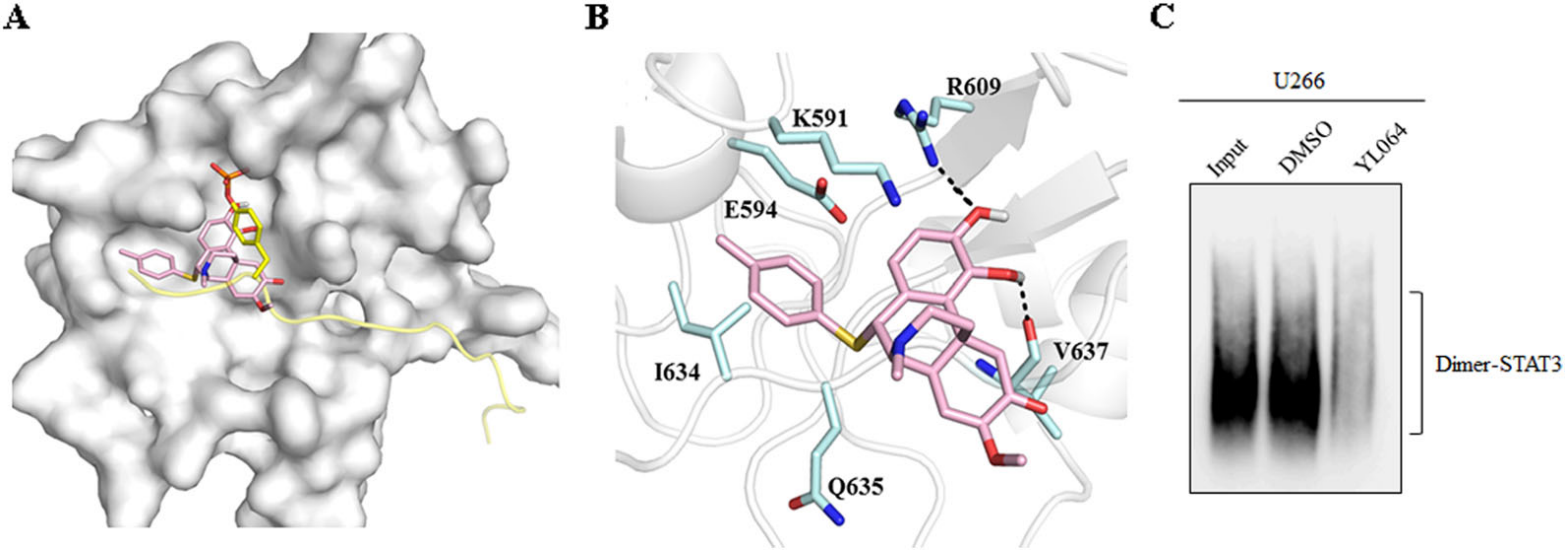
Molecular docking of YL064 with STAT3. **a**, **b** Predicted conformation of YL064 in the pocket of STAT3 SH2 domain (**a**). The molecular surface of STAT3 SH2 domain is shown in white, the phosphotyrosine peptide are colored yellow. YL064 and pTyr705 are shown in pink and yellow sticks, respectively. **b** Residues of STAT3 are shown in cyan sticks and labeled with residue names. The dashed lines in black represent hydrogen bonds. **c** U266 cells were treated with DMSO or YL064 (20 μM) for 24 h, the dimerization of STAT3 was examined in non-denaturating gel

To investigate the structure activity relationship of YL064, a serial of YL064 analogs were synthesized and they showed different potency on U266 cell proliferation (Table S1). For further analyzing the binding mechanisms, a binding model of compound YL064 was generated on the basis of crystal structure of STAT3 β homodimer by molecular docking. It showed that the compound binds to the same sub-pocket that occupied by phosphorylated Tyr705 (Fig. 6a). Similar to pTyr705, which was surrounded by polar residues such as Lys591, Arg609, and Ser611, the hydroxyl group of YL064 also forms polar interactions with Arg609 and the main chain of Val637 (Fig. 6b). It explains that compounds with acetoxyl group at R2 and R3 position have similar activities with those having hydroxyl groups, such as YL064, A6, A7, A8 and A12 vs A18, A19, A20, A21, and A22 (Table S1). The benzyl group of YL064 lies in a cleft that constituted by Glu594 and Ile634 (Figs. 6a, b). Appropriate volume of substituent at R1 position is essential for ligand binding, thus compounds with similar volume of R1 group showed similar bioactivities (Table S1). However, almost little activity was observed even at the concentration of 100 μM for the compounds with small group at R1 position, such as ethyl and propyl group (YL-10-96-A, YL-10-88-A). The IC_50_ value was increased about 2.8-fold for compound A3 (Table S1). Also, substitution at R1 position with larger group can cause the loss of inhibitory activity (A4 and A13) (Table S1). These results indicated that polar interactions between these series of compounds and STAT3 SH2 domain is important. However, shape matching of the compounds to surface around residue Glu594 and Ile634 also play an essential role for their activities, only those with appropriate volume of R1 can clamp their binding and stabilize their conformations. As SH2 domain is important for the dimerization of STAT3, we indeed found that YL064 could disrupt the dimerization of STAT3 in cells (Fig. 6c). Taken together, these data suggest that YL064 may interact with STAT3 in the SH2 domain.

### YL064 suppresses the growth of human MM in vivo

**Fig. 7:**
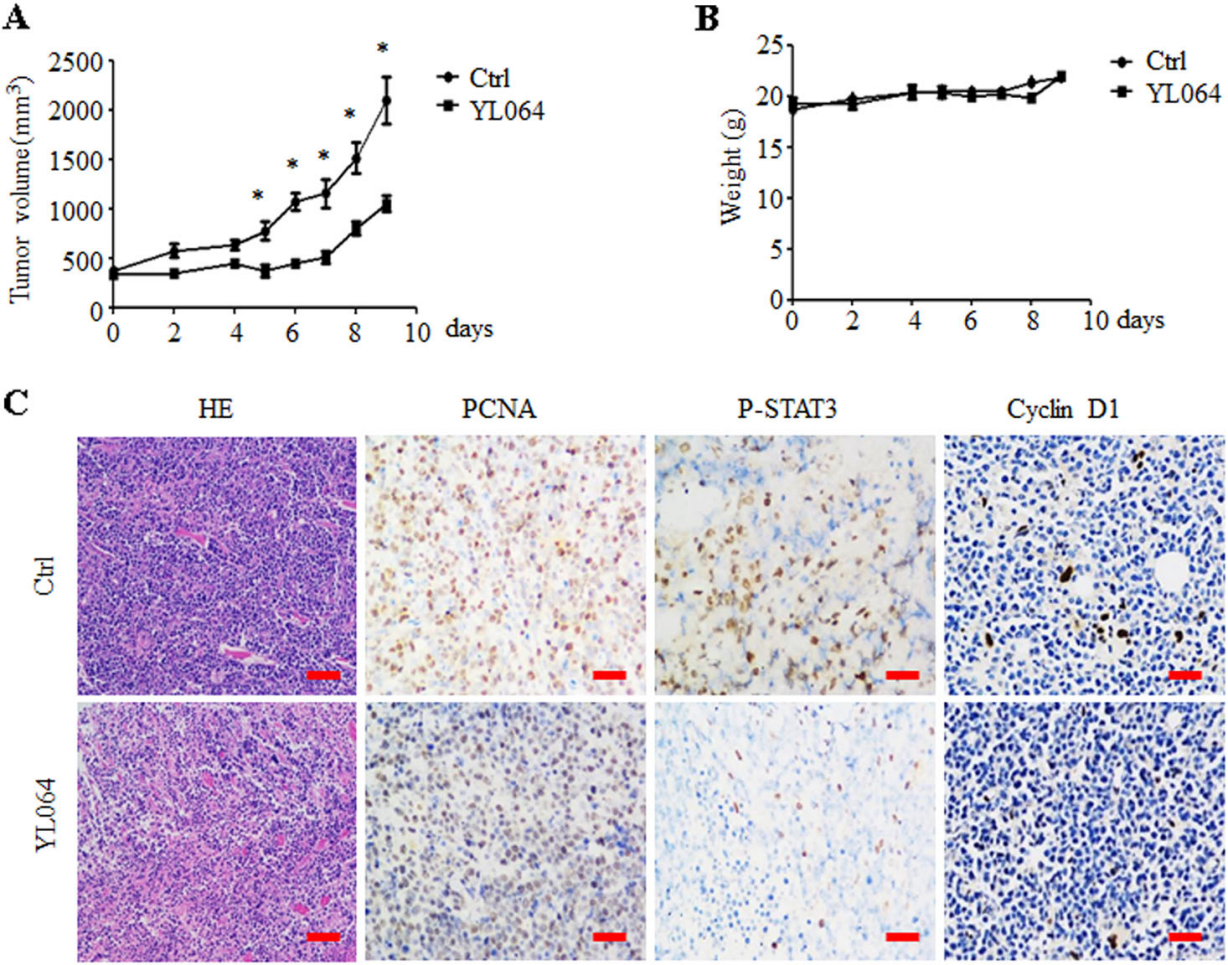
YL064 inhibits myeloma tumor growth in vivo. **a**, **b** Mice bearing MM1.S MM tumors were treated with either YL064 (30 mg/kg; IP) or vehicle daily for 9 days. Tumor volume was measured every 2 days (**a**). Body weight were measured every 2 days (**b**). **c** The hematoxylin–eosin staining analysis of tumors. The indicated proteins were examined by immunohistochemistry (IHC) in tumor tissues (**c**). *p < 0.05, vehicle vs YL064-treated group. Scale bar = 50 μm

The in vivo efficacy of YL064 in a xenograft MM mouse model was also examined. YL064 significantly decreased the tumor burden of MM.1S tumor-bearing mice (Fig. 7a). However, YL064 has no significant effect on the body weight of treated mice (Fig. 7b). Immunohistological analysis indicated that the expression of Proliferating cell nuclear antigen (PCNA), Terminal deoxynucleotidyl transferase dUTP nick end labeling (TUNEL), p-STAT3, and cyclin D1 was decreased upon YL064 treatment (Fig. 7c). These data suggest that YL064 could suppress STAT3 activation and tumor growth in vivo.

## Discussion

In this study, we demonstrated that YL064, a sinomenine derivate, directly interacts with STAT3 in MM cells, and their interaction was involved in YL064-induced cell death. These results indicated that YL064 may be a promising anti-MM compound.

Sinomenine has been used clinically for the treatment of rheumatoid arthritis and other inflammatory diseases^18^. However, high dose of sinomenine are needed to achieve efficacy, which resulted in some side effects and limited its application. Therefore, a series of derivatives was synthesized to improve its potency. Among them,YL064 is the most potent one. Sinomenine has no toxicity to myeloma cells even at 100 μM (Supplemental Fig. S2), whereas YL064 could significantly induce cell death at 10 μM.

YL064 is a promising anti-MM agents. Currently, the combination chemotherapy based on proteasome inhibitors (PIs) or immunomodulatory drugs is the backbone of MM therapy. Bortezomib-containing regimens, including CyBorD (cyclophosphamide, bortezomib, and dexamethasone) and RVD (lenalidomide, bortezomib, and dexamethasone), have been highlighted as frontline regimens for newly diagnosed or relapsed/refractory MM. However, the majority of MM patients relapse due to drug resistance. In the present work, we found that YL064 could effectively induce MM cell death of MM cells in vivo and in vitro. It is known that the bone marrow microenvironment is important in supporting the survival of myeloma cells^7^. Strikingly, YL064 could overcome stromal cell co-culture and exogenous IL-6-mediated protective effect on MM cells through inhibiting STAT3 phosphorylation. Moreover, previous reports have indicated that the extract of tripterygium wilfordii, can inhibit MM cell proliferation and reverse its resistance to bortezomib by interfering with the STAT3 signaling pathway in MM cells^19^. Therefore, YL064 may also be used to overcome bortezomib resistance, which worth further investigation in the future.

We used CETSA method to determine whether YL064 could directly interacts with STAT3 in cells^20,21^. This method utilizes the concept that target proteins usually get stabilized when drug molecules bind in vitro or in cells. Compared with other methods, CETSA is more feasible to perform and demonstrate a deduced interaction between a drug molecule and its targets in cells and animal models^22,23^. Using this method, we found that YL064 directly binds STAT3 in cellular context. Furthermore, we synthesized biotin- and FITC-labeled YL064 to investigate their interaction. Interestingly, biotin-labeled YL064 could pull-down STAT3 but not STAT1 from MM cells and FITC-labeled YL064 colocalized with STAT3 in cells, indicating the interaction of YL064 with STAT3 in cells. More importantly, the binding of biotin-labeled YL064 to recombinant STAT3 could be complete by unlabeled YL064, further supporting the direct interaction between STAT3 and YL064.

The structure of STAT3, begins with a N-terminal domain, a coiled-coil domain, a DNA-binding domain, a linker, and a single SH2 domain (dimerization) and ends with the transactivation domain^24,25^. Our molecular docking studies showed that YL064 may prevents the interaction of STAT3 with phosphorylated tyrosine residues on cytoplasmic receptor kinases through targeting the SH2 domain of STAT3. Currently, several groups are developing STAT3 inhibitor against its SH2 domain^26^. However, there are no such inhibitors in clinical trial. As YL064 is more potent than the clinical used sinomenine in selectively inducing myeloma cell death, and YL064 is well tolerated in mice model, we proposed YL064 as a promising anti-MM agents for clinical trial in the future.

In conclusion, we identified YL064, a sinomenine derivate, could inhibit cell proliferation and induce cell death in myeloma by directly binding STAT3 and inhibiting its activation. Considering the critical role of STAT3 in a variety of cancer cells, the potential therapeutic value of YL064 in other cancers warrants further investigation.

## Materials and methods

### Cells culture

Human MM cell lines U266 and MM1.S, BMSCs (HS-5) and Hela cell line were obtained from American Type Culture Collection (ATCC, Manassas, VA), the MM cell lines were maintained in RPMI-1640 (Gibco, Foster City, CA). Hela and HS-5 cells were cultured in Dulbecco’s modified Eagle’s medium (HyClone, Logan, UT, USA). The medium was supplemented with 10% (v/v) fetal bovine serum and 1% penicillin–streptomycin. They were kept in a humidified atmosphere with 95% air and 5% CO_2_ at 37 °C.

### The synthesis of biotin and FITC-labeled YL064

The synthesis of YL064, biotin-, and FITC-labeled YL064 and YL064 derivatives were detailed in Supplementary materials and methods. The purity of these compounds was determined by high performance liquid chromatography analysis (Anglent, USA).

### Primary MM cells and PBMCs isolation

The mononuclear cells (MNCs) from the bone marrow of MM patients and PBMCs from healthy volunteers were isolated using Ficoll–Hypaque (Pharmacia, Piscataway, NJ, USA) density sedimentation. The CD138-positive cells were selected using EasyStep CD138^+^ magnetic nanoparticles from MNCs (Stem Cell Technologies, Vancouver, BC, Canada) according to the manufacturer’s instructions. All subjects provided informed written consent and the study was conducted in accordance with the Declaration of Helsinki^27^. The study protocol was approved by the Clinical Investigational Reviewing Board of the Shanghai Jiaotong University, China.

### Cell apoptosis

After exposure to YL064 or sinomenine, a number of 2 × 10^5^ U266 and MM1.S cell were subjected for Annexin V/propidium iodide assay (BD Pharmingen, USA). The cell apoptosis ratio were analyzed by FlowJo software (BD Biosciences, San Diego, CA, USA).

### Western blot analysis

After treatment with YL064 or its derivatives, cells were collected and lysed in RIPA lysis buffer (Beyotime, China). The following procedures for western blot have been described previously^28^. The antibodies against phospho-STAT3 (Tyr705), phospho-STAT3 (Ser727), STAT3, STAT5, cyclin D1, Mcl-1, Biotin, β-actin, Vinculin, anti-lamin B used in the present study were purchased from Santa Cruz Biotechnology, USA with a dilution of 1:1000 for usage.

### Luciferase reporter assay

Approximately 1 × 10^6^ Hela cells were transfected with p-STAT3-Luc (Panomics) and SV40-Renilla-Luc (Promega) using Lipofectamine 2000 (Invitrogen,USA). Twenty-four hours later, the cells were pretreated with YL064 for 1 h, and IL-6 (R&D Systems, Minneapolis, MN, USA, 20 ng/ml) was added and incubated for additional 4 h. Then, the cells were collected and lysed in passive lysis buffer (Promega) for 15 min, the transcriptional activity of STAT3 was determined using a luciferase reporter assay system (Promega), according to the manufacturer’s instructions.

### EMSA

A number of 4 × 10^5^ U266 cells were pretreated with YL064 for 24 h, then the cells were co-cultured with IL-6 (20 ng/ml) for 30 min. Next, the cytoplasmic and nuclear proteins were separated using the NE-PER nuclear and cytoplasmic extraction reagent (Pierce Biotech, Rockford, IL), according to the manufacturer’s protocol. The nuclear extracts (5 μg) were preincubated in a binding buffer containing 1 μg of poly (dI:dC) (Amersham Biosciences), followed by addition biotin-labeled oligonucleotide probe containing STAT3 element. The wild-type oligonucleotides (5′-GATCCTTCTGGGAATTCCTAGATC-3′) and (3′-CTAGGAAGACCCTTAAGGATCTAG-5′) were biotin labeled using a biotin 3′ end DNA labeling kit (Pierce Biotech). The probe or competitors used were prepared by annealing the sense and antisense synthetic oligonucleotides. Reaction mixtures were then separated on a 4% native polyacrylamide gel, and shifted bands that corresponded to protein/DNA complexes were captured by a horseradish peroxidase-based detection system. To examine the specificity of the STAT3 element probe, unlabeled competitor oligonucleotides were preincubated with nuclear extracts for 15 min before incubation with probes. We used antibodies specific for STAT3 to identify STAT3 protein in the DNA protein complex. These antibodies were incubated with the nuclear extracts for 25 min at room temperature before adding loading buffer.

### Real time-polymerase chain reaction PCR (RT-PCR)

Total RNA was extracted using the TRIzol (Invitrogen) method. Complementary DNA was transcribed using a reverse transcription kit (Promega, Japan) according to manufacturer’s instructions. The expression of cyclin D1, Mcl-1, and GAPDH was detected by real-time (RT-PCR) using the ABI PRISM 7900 system (Applied Biosystems, USA). The following RT-PCR cycling conditions were used: 95 °C for 5 min; 40 cycles at 95 °C for 5 s, and at 60 °C for 30 s; and then elongation was performed at 60 °C for 10 min. The relative mRNA expression of the target genes was analyzed by the 2^−ΔΔCt^ method, and GAPDH served as an internal control. The primers for cyclin D1 were: forward: 5′-GCGTACCCTGACACCAATCTC-3′, reverse: 5′-CTCCTCTTCGCACTTCTGCTC-3′. The primers for Mcl-1 were: forward: 5′-GGGCAGGATTGTGACTCTCATT-3′, reverse: 5′-GATGCAGCTTTCTTGGTTTATGG-3′. The primers for GAPDH were: forward: 5′-CATCAAGAAGGTGGTGAAGC-3′, reverse: 5′-ACCACCCTGTTGCTGTAG-3′.

### Immunofluorescence assay

The U266 or MM cells were cultured on slides and fixed with 0.3% Triton X-100. After permeabilization with 100% cold methanol and blocking with 2% (w/v) bovine serum albumin, the cells were incubated STAT3 antibody (1:100 dilution), YL064 (1:100 dilution), or FITC-YL064 (1:100 dilution) overnight at 4 °C. The slides were stained with the secondary antibodies counterstained with 4,6-diamidino-2-phenylindole (Molecular Probes). Fluorescence signals were detected on a Bio-Rad MRC-1024 laser scanning confocal microscope (Olympus America).

### Cell co-culture

For the cell co-culture experiment, 5 × 10^5^ HS-5 cells were seeded in a 10-cm Petri dish. Twenty-four hours later, 5 × 10^5^ MM cells were added into the dish. After incubation for another 24 h, cells were collected for further experiments.

### CETSA

U266 cells were used for CETSA and the amount for YL064 incubation was 100 μM. The procedure of CETSA has been described previously^29^.

### Pull-down and analysis of YL064-bound proteins

U266 cell lysates were incubated with biotin or biotin-YL064 in the absence or presence of unlabeled YL064 overnight at 4 °C, and the streptavidin bead-bound proteins were separated by sodium dodecyl sulfate-polyacrylamide gel electrophoresis (SDS-PAGE) and examined by western blot.

### Preparation of recombinant wild-type STAT3

Human STAT3 were cloned into pET28a vector containing a 6×His tag at the N-terminal region. These proteins were expressed in the *Escherichia coli* strain BL21 and purified.

### Native gel PAGE

Cell extracts containing native proteins were prepared using ice-cold isotonic buffer [20 mmol/L Tris (pH 7.0), 150 mmol/L NaCl, 6 mmol/L MgCl_2_, 0.8 mmol/L Phenylmethanesulfonyl fluoride, and 20% glycerol]. Lysates were homogenized using a 27-gauge syringe and then cleared by centrifugation at 13,000 rpm for 30 min at 4 °C. Native PAGE analysis was carried out by loading 10 μg samples onto 6% SDS-free, PAGE gels. Proteins were transferred to Polyvinylidene fluoride membranes (Millipore) and immunoblotted with specific antibody as described for western blot analysis.

### Molecular docking

The binding modes between STAT3 and the compounds were analyzed using software AutoDock4.2^30,31^. The X-ray crystal structure of the STAT3 β homodimer bound to DNA solved at 2.25-Å resolution was retrieved from protein data bank (PDB code: 1BG1^31^) for docking simulation. DNA, all crystallographic water, and other small molecules were removed, the pTyr (pY) peptide was extracted. Molecules were docked to its SH2 domain. The binding site was covered by preparing a 60 × 60 × 60 size of grid box with grid spacing of 0.375 Å of spacing between grid points. The coordinate center of pTyr705 was defined as the center of the box. The Lamarckian genetic algorithm was applied to obtain the protein–ligand binding free energies and the bound spatial conformations of compounds. Protein was considered rigid and small molecules were flexible during docking process. The docking parameters were set as follows: ga_pop_size, 150, ga_run, 100, other parameters were set to the software’s default values. The resulted conformations were clustered according to the 2.0 Å root-mean-square deviation criteria and ranked by binding free energy. Finally, the best binding conformations of the ligands were selected by taking account of the predicted binding free energy for further analysis.

### Xenograft model

Female BALB/c nu/nu mice aged 4–6 weeks, obtained from Silaike (Shanghai, China), were kept under specific-pathogen-free conditions and used according to the Shanghai Medical Experimental Animal Care guidelines.

A number of 3 × 10^7^ MM1.S cells were injected subcutaneously with matrix gel into the right hips. When tumor was palpable, the mice were divided into a vehicle and YL064 groups (*n* = 5/group). Vehicle (8% dimethyl sulfoxide, 10% 1,2-Propanediol, 10% Kolliphor ELP and 72% normal saline) and YL064 (30 mg/kg, intraperitoneal for 8 days). Body weight was monitored daily. Tumor sizes were measured with calipers, and the tumor volumes were calculated by the formula: tumor volume = 0.5 × *a* × *b*^2^, where “*a*” is the length and “*b*” is the width. Animal study protocols were approved by the Institutional Animal Care and Use Committee of Shanghai Jiao Tong University School of Medicine.

### Histology

The expression of PCNA, cyclin D1, TUNEL, and p-STAT3 in tumor tissues was determined using immunohistochemistry staining, the procedure of which has been previously described^29^.

### Statistical analysis

The data were expressed as mean ± SEM. The paired samples *t*-test was applied to measure the significance in the cell line experiments using the PASW Statistic 18 software (IBM, Armonk, New York, USA). The Wilcoxon signed-rank test was used in the in vivo experiments, to determine the significance of the between-group differences. A two-way *P-*value <0.05 was considered statistically significant.

## Electronic supplementary material

Supp Figure 1

Supp Figure 2

Supplementary files
