# Supplementary figures and images for: YL064 directly inhibits STAT3 activity to induce apoptosis of multiple myeloma cells
Source: Cell Death Discov. 2018 Oct 3;4:44. doi: 10.1038/s41420-018-0108-8 (PMC6170385; doi:10.1038/s41420-018-0108-8)

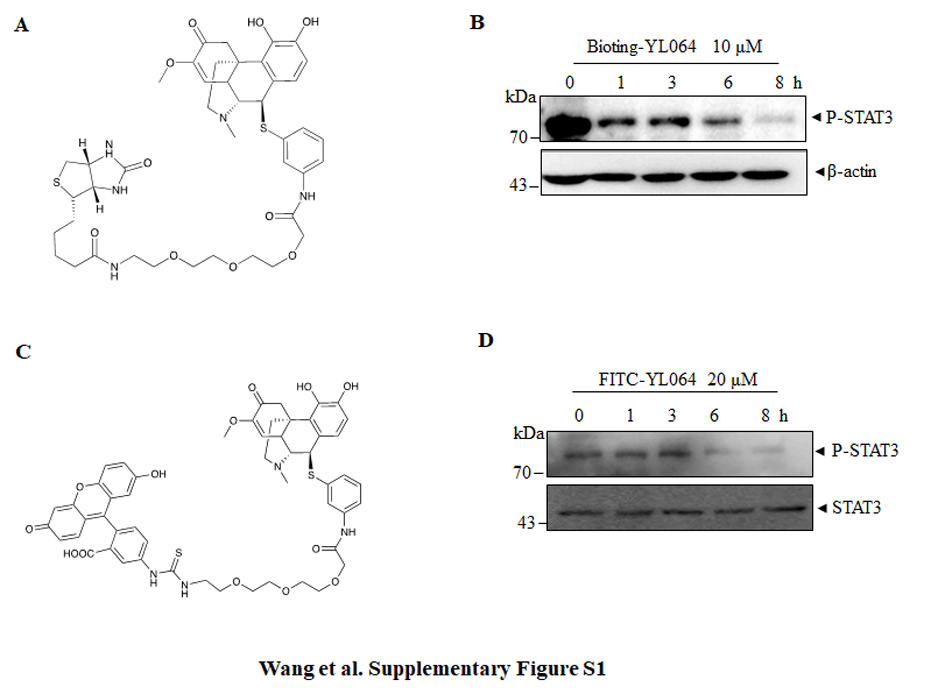

Supplement: Supplementary file 1 — Supp Figure 1 [file 41420_2018_108_MOESM1_ESM.tif]

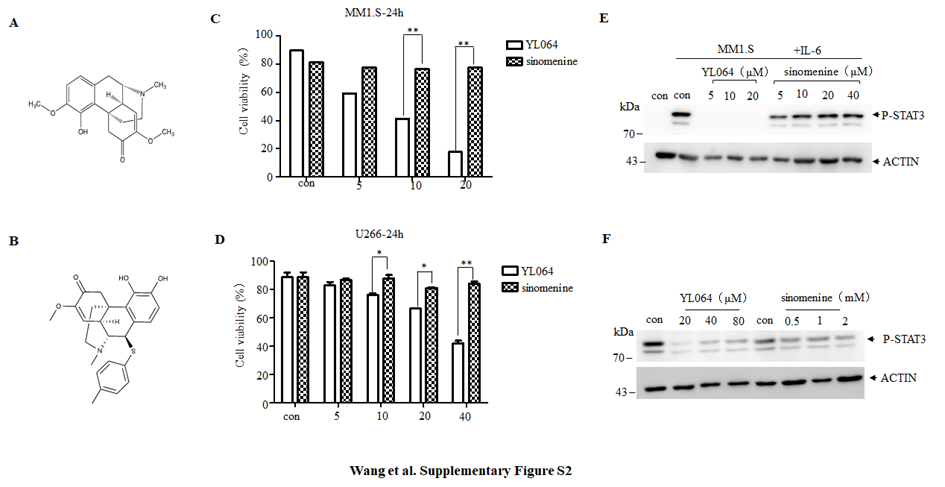

Supplement: Supplementary file 2 — Supp Figure 2 [file 41420_2018_108_MOESM2_ESM.tif]
