# Supplementary material for: YL064 directly inhibits STAT3 activity to induce apoptosis of multiple myeloma cells
Source: Cell Death Discov. 2018 Oct 3;4:44. doi: 10.1038/s41420-018-0108-8 (PMC6170385; doi:10.1038/s41420-018-0108-8)
Supplement: Supplementary file 3 — Supplementary files [file 41420_2018_108_MOESM3_ESM.docx]

**YL064 induces apoptosis in multiple myeloma cells in vitro and in vivo through directly targeting STAT3**

Yingying Wang^1,3#^, Linlin Wu^2#^, Haiyan Cai^1#^, Hu Lei^1^, Chun-Min Ma^1^, Li Yang^1^, Hanzhang Xu^1^, Qi Zhu^3*^, Zhujun Yao^2*^, Yingli Wu^1*^

**Supplementary Table S1. Effect of YL064 analogs on the proliferation of myeloma cells.**

| Compounds | R1 | R2 | R3 | Myeloma cell inhibition  IC_50_ (μM) |
| --- | --- | --- | --- | --- |
| A1 | -CH2CH3 | OH | OH | >100 |
| A2 | -CH2CH2CH3 | OH | OH | >100 |
| A3 | -CH2(CH2)_2_CH3 | OH | OH | 78.3 |
| A4 |  | OH | OH | >100 |
| YL064 |  | OH | OH | 28 |
| A5 |  | OH | OH | 33.3 |
| A6 |  | OH | OH | 24.9 |
| A7 |  | OH | OH | 23.0 |
| A8 |  | OH | OH | 21.6 |
| A9 |  | OH | OH | 47.4 |
| A10 |  | OH | OH | 28.5 |
| A11 |  | OH | OH | 24.8 |
| A12 |  | OH | OH | 21.7 |
| A13 |  | OH | OH | >100 |
| A14 |  | OH | OH | 34.3 |
| A15 |  | OH | OH | 25.3 |
| A16 |  | OH | OH | 22.4 |
| A17 |  | OH | OH | 58.0 |
| A18 |  | OAc | OAc | 28.1 |
| A19 |  | OAc | OAc | 23.7 |
| A20 |  | OAc | OAc | 24.4 |
| A21 |  | OAc | OAc | 28.6 |
| A22 |  | OAc | OAc | 25.6 |
| A23 |  | OAc | OAc | 24.4 |

**Supplementary Material and Methods**

**Synthesis of YL064 and its analogs A1-A17 (Table S1)**

All the sinomenine derivatives in Table S1 were synthesized through a two-step or three-step procedure starting from natural sinomenine via a benzoquinone intermediate.

**Benzoquinone intermediate.**^[[1]](#footnote-1)^ A solution of sinomenine hydrochloride salt (21 g, 57.4 mmol, 1 equiv) in water (420 mL) was treated with (diacetoxyiodo)benzene (DIB, 21 g, 65.2 mmol, 1.1 equiv) at room temperature for 1 hour. The mixture was then basified with sat. aq. NaHCO_3_ and extracted with CH_2_Cl_2_ (100 mL x 3). The combined organic layers were combined, washed with water and brine, dried over Na_2_SO_4_, filtered, and concentrated. The residue was purified by flash column chromatography on silica gel (CH_2_Cl_2_/acetone = 1/1) to afford a bright yellow solid (12 g, 67%). m.p. 187-188 ^o^C (dec.); [α]_D_^25^ = 563.3 (*c* 0.80, CHCl_3_).

**Preparation of YL064 and sinomenine derivatives A1-A17 (R^2^ = R^3^ = OH)**. A solution of the above benzoquinone intermediate (0.5 g, 1.60 mmol) in DCM (15 mL) was treated with the corresponding thiol (R^1^SH, 1.76 mmol, 1.1 equiv) at 0 ^o^C to room temperature until completion of the reaction. Petroleum ether was then added to precipitate the product. The resulting solid was filtered, washed with petroleum ether and dried in vacuum to afford the sinomenine derivatives. **YL064** (R^1^ = *p*-MeC_6_H_4_-, R^2^ = R^3^ = OH): light yellow solid, 79% yield. m.p. 224-225 ^o^C (dec.); [α]_D_^26^ 7.0 (*c* 0.50, CHCl_3_-CH_3_OH = 10:1); ^1^H NMR (DMSO-d_6_, 400 MHz): δ 9.47 (1H, s), 8.18 (1H, s), 7.57 (2H, d, *J* = 8.0 Hz), 7.25 (2H, d, *J* = 7.6 Hz), 7.02 (1H, d, *J* = 8.4 Hz), 6.67 (1H, d, *J* = 8.0 Hz), 6.27 (1H, d, *J* = 2.0 Hz), 4.47 (1H, s), 4.18 (1H, d, *J* = 15.2 Hz), 3.53 (3H, s), 3.11-3.18 (1H, m), 2.99 (1H, brs), 2.39 (1H, d, *J* = 15.2 Hz), 2.33 (3H, s), 2.24-2.32 (1H, m), 1.93 (3H, s), 1.80 (1H, td, *J* = 4.4, 10.8 Hz), 1.62-1.76 (2H, m); ^13^C NMR (DMSO-d_6_, 75 MHz): δ 192.4, 149.4, 143.9, 137.3, 132.7, 132.0, 129.8, 128.3, 123.7, 120.3, 117.8, 113.3, 59.7, 53.9, 48.4, 45.9, 44.7, 43.6, 41.7, 35.5, 20.6; IR (KBr): *v_max_* 3325.9, 2940.9, 2831.0, 1666.8, 1619.8, 1491.3, 1291.3, 1202.0, 1151.8, 1092.9, 808.1, 497.4 cm^-1^; HRMS (ESI, m/z) calcd. for C_25_H_28_N_1_O_4_S_1_ (M+H^+^): 438.1734, Found: 438.1742.

**Preparation of sinomenine derivative diacetates A18-A23 (R^2^ = R^3^ = OAc)**. A solution of catechol-type derivative (0.91 mmol) in pyridine (4 mL) was treated with acetic anhydride (1.82 mmol, 2 equiv) at 0 ^o^C to room temperature until completion of the reaction. The reaction mixture was concentrated and purified by column chromatography (DCM/CH_3_OH 50/1) to afford the corresponding diacetate derivatives. **YL064** **diacetate** (**A21**, R^1^ = *p*-MeC_6_H_4_-, R^2^ = R^3^ = OAc): white solid, 90% yield. m.p. 206-207 ^o^C; [α]_D_^26^ -42.9 (*c* 0.40, CHCl_3_); ^1^H NMR (CDCl_3_, 400 MHz): δ 7.67 (1H, d, *J* = 8.8 Hz), 7.50 (2H, d, *J* = 8.0 Hz), 7.16-7.24 (3H, m), 6.28 (1H, d, *J* = 2.0 Hz), 4.41 (1H, s), 3.77 (1H, d, *J* = 16.0 Hz), 3.64 (3H, s), 3.43 (1H, d, *J* = 3.2 Hz), 3.08 (1H, s), 2.51 ( 1H, d, *J* = 16.0 Hz), 2.38 (3H, s), 2.37 (3H, s), 2.32-2.37 (1H, m), 2.22 (3H, s), 2.04 (3H, s), 1.94 (1H, dt, *J* = 2.8, 12.0 Hz), 1.84 (1H, dt, *J* = 4.4, 12.4 Hz), 1.56 (1H, d, *J* = 12.0 Hz); ^13^C NMR (CDCl_3_, 100 MHz): δ 191.7, 168.0, 167.5, 150.9, 142.2, 141.0, 138.5, 136.3, 133.2, 132.0, 130.9, 130.2, 128.0, 121.8, 116.8, 60.7, 54.9, 50.2, 46.0, 45.7, 44.3, 42.1, 40.8, 37.5, 21.2, 21.0, 20.9; IR (KBr): *v_max_* 3418.8, 2929.8, 1774.5, 1689.6, 1622.1, 1472.0, 1372.9, 1262.9, 1203.3, 1177.8, 1144.3, 1088.3, 1013.3, 929.7, 812.0, 500.0 cm^-1^;. MS (MALDI, m/z): 522.1 (M+H)^+^, Anal. Calcd. for C_29_H_31_NO_6_S requires C: 66.77; H: 5.99; N: 2.69, found C: 66.86; H: 6.02; N: 2.79.

**Synthesis of biotin-YL064**

A mixture of biotin (1.45 g, 5.92 mmol), HATU (1-[bis(dimethylamino)methylene]-1H-1,2,3-triazolo[4,5-b]pyridinium 3-oxid hexafluorophosphate, 2.48 g, 6.51 mmol) and DIPEA (N,N-diisopropylethylamine, 842 mg, 1.13 mL, 6.51 mmol) in anhydrous DMF (30 mL) was stirred under nitrogen atmosphere at 0 ^o^C for 1 hour. The free amine **S1** (1.55 g, 5.91 mmol) in DMF (20 mL) was then added, and the reaction was allowed to warm to rt and stirred for additional 12 hours. Ice-water (100 mL) was added to quench the reaction, and the mixture was extracted with ethyl acetate (100 mL x 5). The organic phases were combined and dried over anhydrous Na_2_SO_4_, filtered and concentrated. The residue was purified on silica gel chromatography (DCM/MeOH = 40/1 to 20/1) to give **S2** (2.56 g, 88%). [α]_D_^25^ = +32.0 (*c* 0.3, CHCl_3_); ^1^H NMR (400 MHz, CDCl_3_): δ 6.85 (t, *J* = 5.6 Hz, 1H), 6.52 (s, 1H), 5.59 (s, 1H), 4.51 (dd, *J* = 7.8, 4.8 Hz, 1H), 4.31 (dd, *J* = 8.0, 4.6 Hz, 1H), 4.00 (s, 2H), 3.74 – 3.59 (m, 8H), 3.56 (t, *J* = 5.0 Hz, 2H), 3.42 (p, *J* = 4.9 Hz, 2H), 3.13 (td, *J* = 7.4, 4.5 Hz, 1H), 2.90 (dd, *J* = 12.8, 4.9 Hz, 1H), 2.74 (d, *J* = 12.8 Hz, 1H), 2.23 (t, *J* = 7.4 Hz, 2H), 1.80 – 1.59 (m, 4H), 1.46 (m, 11H); IR (KBr): *v*_max_ 3292.5，2927.1，2856.4，1747.1，1705.9，1644.8，1552.7，1461.8，1368.4，1260.4，1125，1021.9，842.9，800.8，727.7，699.7，603.5 cm^-1^; HRMS (ESI, m/z) calcd. for C_22_H_39_N_3_O_7_S (M+H^+^):490.2581, Found: 490.2581.

To a solution of **S2** (250 mg, 0.50 mmol) in anhydrous DCM (10 mL) was added CF_3_COOH (2 mL) and Et_3_SiH (0.8 mL) at 0 ^o^C. The reaction was stirred at rt until the reactant was consumed. The whole mixture was concentrated under vacuum to dryness. The resulting residue was treated with HATU (209 mg, 0.55 mmol) and DIPEA (0.1 mL, 77 mg, 0.6 mmol) in dry DMF (5 mL) at rt for 2 hours, until **A17** (220 mg, 0.50 mmol) in DMF (5 mL) was added at 0 ^o^C. After completion of the reaction, cold water (20 mL) was added. The whole mixture was concentrated under vacuum. The residue was purified by preparative TLC (DCM/MeOH = 8/1) to afford **biotin-YL064** (225 mg, 51%). [α]_D_^25^ = +31.33 (*c* 0.3, MeOH); ^1^H NMR (400 MHz, DMSO-*d*_6_): δ 9.82 (s, 1H), 9.56 (s, 1H), 8.23 (s, 1H), 8.02 (s, 1H), 7.86 (d, *J* = 5.7 Hz, 1H), 7.60 (d, *J* = 3.9 Hz, 1H), 7.37 (d, *J* = 4.9 Hz, 2H), 6.97 (d, *J* = 8.2 Hz, 1H), 6.68 (d, *J* = 8.2 Hz, 1H), 6.39 (d, *J* = 21.7 Hz, 2H), 6.23 (d, *J* = 2.2 Hz, 1H), 4.54 (s, 1H), 4.29 (t, *J* = 6.3 Hz, 1H), 4.24 – 4.04 (m, 5H), 3.67 (t, *J* = 4.6 Hz, 2H), 3.61 (d, *J* = 5.0 Hz, 3H), 3.53 (h, *J* = 5.0 Hz, 10H), 3.17 (dd, *J* = 7.9, 4.4 Hz, 5H), 3.07 (dt, *J* = 10.2, 5.4 Hz, 1H), 3.00 (s, 1H), 2.80 (dd, *J* = 12.4, 5.0 Hz, 1H), 2.57 (d, *J* = 12.4 Hz, 1H), 2.40 (d, *J* = 15.2 Hz, 1H), 2.32 (d, *J* = 10.6 Hz, 1H), 2.11 – 1.93 (m, 6H), 1.82 (s, 1H), 1.71 (d, *J* = 9.8 Hz, 2H), 1.66 – 1.37 (m, 5H), 1.27 (dq, *J* = 21.0, 6.5 Hz, 5H); ^13^C NMR (101 MHz, DMSO-*d*_6_): δ 192.59 , 172.20 , 168.63 , 149.55 , 144.21 , 144.11 , 139.13 , 136.02 , 129.61 , 128.19 , 127.15 , 123.80 , 122.90 , 120.33 , 118.82 , 118.81 , 117.77 , 113.54 , 70.36 , 70.24 , 69.76 , 69.64 , 69.54 , 69.19 , 61.07 , 60.06 , 59.22 , 55.45 , 54.05 , 48.62 , 48.47 , 46.08 , 44.77 , 43.41 , 41.94 , 38.89 , 38.45 , 35.12 , 28.22 , 28.06 , 25.28 ; IR (KBr): *v*_max_ 3443.6，2922.9，1632.9，1555.1，1532.7，1384.7，1311.2，1121.3，1030.8，781.3 cm^-1^; HRMS (ESI, m/z) calcd. for C_42_H_55_N_5_O_10_S_2_ (M+H^+^):854.3463, Found: 854.3462.

**Synthesis of FITC-YL064**

A mixture of **A17** (101 mg, 0.33 mmol), HATU (136 mg, 0.36 mmol) and DIPEA (46.5 mg, 0.36 mmol) in dry DMF (3 mL) was stirred at rt for 2 hours. Then, the acid **S3** (101 mg, 0.33 mmol) in dry DMF (3 mL) was added at 0 ^o^C. The mixture was stirred at rt for 12 hours until cold water (6 mL) was added to quench the reaction. The whole mixture was concentrated to dryness under vacuum. The residue was purified by preparative TLC (DCM/MeOH = 9/1) to give **S4** (100 mg, 45%). [α]_D_^25^ = +12.67 (*c* 0.3, CHCl_3_); ^1^H NMR (400 MHz, CDCl_3_): δ 8.11 (s, 1H), 7.45 – 7.31 (m, 3H), 7.07 (d, *J* = 8.3 Hz, 1H), 6.69 (d, *J* = 8.3 Hz, 1H), 6.28 (d, *J* = 2.3 Hz, 1H), 5.10 (s, 1H), 4.55 (s, 1H), 4.36 (d, *J* = 15.4 Hz, 1H), 4.16 (s, 2H), 3.85 – 3.55 (m, 12H), 3.49 (q, *J* = 4.4, 3.6 Hz, 3H), 3.26 (q, *J* = 5.4 Hz, 2H), 3.14 (s, 1H), 2.43 (d, *J* = 16.1 Hz, 2H), 2.14 (s, 3H), 2.01 (s, 1H), 1.42 (s, 9H); ^13^C NMR (101 MHz, CDCl_3_): δ 194.46 , 169.06 , 156.29 , 150.54 , 144.38 , 143.65 , 138.23 , 136.78 , 129.87 , 128.71 , 124.29 , 122.95 , 121.32 , 119.70 , 117.37 , 114.13 , 79.62 , 77.36 , 71.28 , 70.50 , 70.26 , 70.10 , 70.00 , 61.16 , 54.90 , 49.09 , 46.76 , 45.25 , 44.35 , 42.18 , 40.37 , 35.85 , 28.52 , 17.97 ; IR (KBr): *v*_max_ 3445.1，2925.1，1686.2，1628.2，1583.2，1530.3，1476.7，1397.9，1288.2，1202.4，1149.3，1121.5，1100.9，845.7，559.1 cm^-1^; HRMS (ESI, m/z) calcd. for C_37_H_49_N_3_O_10_S (M+H^+^): 728.3211, Found: 728.3214.

A solution of S4 (90 mg, 0.123 mmol) in anhydrous DCM (2 mL) was treated with CF_3_COOH (0.4 mL) and stirred at rt until completion of the reaction. The whole mixture was concentrated to dryness in vacuum. The mixture of the resulting residue, the commercially available reagent **S5** (50 mg, 0.124 mmol), and DIPEA (32.3 mg, 0.25 mmol) in dry DMF (5 mL) was stirred at rt for 12 hours, and then concentrated to dryness under vacuum. The residue was purified by preparative TLC (DCM/MeOH = 5/1) and gel chromatography (CHCl_3_/MeOH = 1/1), successively, affording **FITC-YL064** (58 mg, 46%). [α]_D_^25^ = +38.0 (*c* 0.1, MeOH); ^1^H NMR (400 MHz, CDCl_3_/MeOD=1/1): δ 8.10 (d, *J* = 2.1 Hz, 1H), 8.05 (s, 1H), 7.86 (d, *J* = 7.3 Hz, 1H), 7.51 (ddd, *J* = 6.5, 4.7, 2.8 Hz, 1H), 7.37 (d, *J* = 5.0 Hz, 2H), 7.12 (dd, *J* = 19.6, 8.3 Hz, 2H), 6.81 – 6.64 (m, 6H), 6.55 (ddd, *J* = 9.5, 7.3, 2.4 Hz, 2H), 6.33 (d, *J* = 2.3 Hz, 1H), 4.60 (s, 2H), 4.18 (s, 2H), 3.77 (ddt, *J* = 24.3, 10.1, 4.5 Hz, 13H), 3.65 (d, *J* = 3.9 Hz, 4H), 3.48 (dd, *J* = 8.1, 3.4 Hz, 1H), 3.08 (s, 1H), 2.54 – 2.44 (m, 1H), 2.43 – 2.34 (m, 1H), 2.16 (s, 3H), 2.13 – 2.03 (m, 1H), 1.94 (d, *J* = 12.8 Hz, 1H), 1.78 (td, *J* = 12.8, 4.7 Hz, 1H), 1.39 (dd, *J* = 7.0, 2.0 Hz, 1H), 1.20 (t, *J* = 7.0 Hz, 1H); ^13^C NMR (101 MHz, CDCl_3_/MeOD=1/1): δ 194.23, 169.54, 168.69, 149.59, 143.42, 137.56, 135.40, 129.07, 128.82, 128.80, 127.87, 123.50, 121.57, 120.42, 119.17, 116.71, 112.92, 102.02, 101.97, 77.11, 76.98, 76.78, 76.46, 70.07, 69.75, 69.70, 69.54, 60.20, 60.19, 53.87, 48.29, 48.15, 48.08, 47.94, 47.86, 47.72, 47.65, 47.44, 47.23, 47.01, 45.89, 43.51, 43.35, 40.72, 34.38, 16.77, 15.87, 15.68, 15.49; IR (KBr): *v*_max_ 3442.6，2950.4，2865.7，2843.7，1636.7，1456，1054.7，1032.5，1014.8 cm^-1^; HRMS (ESI, m/z) calcd. for C_53_H_52_N_4_O_13_S_2_ (M+H^+^):1017.3045, Found: 1017.3049.

**Supplementary Figure legends**

**Supplementary Fig. S1**. a&c. Chemical structure of biotin-YL064 (a) and FITC-YL064 (c). b&d. U266 cells were treated with 10 μM of biotin-YL064 (b) or 20 μM FITC-YL064 (d) for the indicated hours. Then cells were collected and subjected to western blot analyses with specific antibodies directed against p-STAT3 (Tyr705). Each experiment was repeated at least three times.

**Supplementary Fig. S2**. Chemical structure of sinomenine (a), and YL064 (b). c-d. MM1.S (c) and U266 (d) cells were treated with the indicated concentrations of YL064 or sinomenine for 24 h. the cell viability were measured by CCK8 assay. *p<0.05. E-F. MM1.S (e) and U266 (f) cells were treated with the indicated concentrations of YL064 or sinomenine for 8 h. Cells were collected and subjected to western blot analyses with specific antibodies directed against p-STAT3 (Tyr705). Each experiment was repeated at least three times.

1. Lou, Y.-T.; Ma, L.-Y.; Wang, M.; Yin, D.; Zhou, T.-T.; Chen, A.-Z.; Ma, Z.; Bian, C.; Wang, S.-z.; Yang, Z.-Y.; Sun, B.; Yao, Z.-J. Regio- and stereoselective C10b-H functionalization of sinomenine: an access to more potent immunomodulating derivatives. *Tetrahedron* **2012**, *68*, 2172-2178. [↑](#footnote-ref-1)
